# Editorial

**Published:** 1877-03

**Authors:** 


### Editorial.

The Chicago Medical Press Association has concluded henceforth to take the publication of the JOURNAL AND EXAMINER in its own charge. To this end the editorial corps has been enlarged by the addition of an assistant editor, in order to enable the editors to conduct the publishing department in addition to the discharge of their former duties.

Dr. E. F. Ingals was elected to the position of assistant editor, and will have charge of the business affairs of the JOURNAL AND EXAMINER.

The publishing and editorial office now is located at 188 SOUTH CLARK ST. All letters or communications of a business character, relating to the publication, advertisements, subscriptions, etc., should be sent to the assistant editor, while all articles and communications intended for publication, also all books and pamphlets for review, should be addressed to the editor.

It is hardly necessary to add that every effort will be made to render the JOURNAL AND EXAMINER worthy of the patronage and commendation of the profession.

We earnestly appeal to every member of the profession to come to our aid with original contributions, and to use what influence he can exert to extend the circulation of our periodical.

We may say to our friends and patrons that our prospects are such as to justify us in promising improvement in every respect. Our pecuniary basis is substantial; we are surrounded by liberal, intelligent and energetic *confrères*, who are devoted to the success of our periodical; we have a large foreign and domestic exchange list; in fact, everything, so far as we can judge, gives the assurance of continued prosperity.

The confusion in our affairs caused by the misfortune of our late publishers, has undoubtedly prevented many who would otherwise have become subscribers from remitting for the subscription. But now, since perfect order has been restored by the complete re-organization of the publishing department, we hope that our friends will no longer hesitate to forward their subscriptions for the JOURNAL AND EXAMINER.

